# Outcomes of children exposed *in utero* to chemotherapy for breast cancer

**DOI:** 10.1186/s13058-014-0500-0

**Published:** 2014-12-30

**Authors:** Rashmi K Murthy, Richard L Theriault, Chad M Barnett, Silvia Hodge, Mildred M Ramirez, Andrea Milbourne, Sue A Rimes, Gabriel N Hortobagyi, Vicente Valero, Jennifer K Litton

**Affiliations:** Department of Breast Medical Oncology, The University of Texas MD Anderson Cancer Center, 1515 Holcombe Boulevard, Box 1354, Houston, TX 77030 USA; Division of Pharmacy, The University of Texas MD Anderson Cancer Center, 1515 Holcombe Boulevard, Houston, TX 77030 USA; Department of Obstetrics/Gynecology, The University of Texas Health Science Center, 6431 Fannin Street, Houston, TX 77030 USA; Department of Gynecologic Oncology and Reproductive Medicine, The University of Texas MD Anderson Cancer Center, 1515 Holcombe Boulevard, Unit 1362, Houston, TX 77030 USA

## Abstract

**Introduction:**

The incidence of breast cancer diagnosed during pregnancy is expected to increase as more women delay childbearing in the United States. Treatment of cancer in pregnant women requires prudent judgment to balance the benefit to the cancer patient and the risks to the fetus. Prospective data on the outcomes of children exposed to chemotherapy *in utero* are limited for the breast cancer population.

**Methods:**

Between 1992 and 2010, 81 pregnant patients with breast cancer were treated in a single-arm, institutional review board–approved study with 5-fluorouracil, doxorubicin, and cyclophosphamide (FAC) in the adjuvant or neoadjuvant setting. Labor and delivery records were reviewed for each patient and neonate. In addition, the parents or guardians were surveyed regarding the health outcomes of the children exposed to chemotherapy *in utero*.

**Results:**

In total, 78% of the women (or next of kin) answered a follow-up survey. At a median age of 7 years, most of the children exposed to chemotherapy *in utero* were growing normally without any significant exposure-related toxicity or health problems. Three children were born with congenital abnormalities: one each with Down syndrome, ureteral reflux or clubfoot. The rate of congenital abnormalities in the cohort was similar to the national average of 3%.

**Conclusions:**

During the second and third trimesters, pregnant women with breast cancer can be treated with FAC safely without concerns for serious complications or short-term health concerns for their offspring who are exposed to chemotherapy *in utero*. Continued long-term follow-up of the children in this cohort is required.

**Trial registration:**

ClinicalTrials.gov Identifier: NCT00510367. Other Study ID numbers: ID01-193, NCI-2012-01578. Registration date: 31 July 2007.

## Introduction

Breast cancer is the most common malignancy associated with pregnancy; however, only 0.2% to 2.9% of all breast cancers occur during pregnancy [1,2]. As women are delaying childbearing, the incidence of breast cancer during pregnancy is increasing [3-5]. Management of breast cancer during pregnancy requires an intricate balance between using multiple modalities to effectively treat the mother and minimizing the potential toxicities for the fetus [6]. The factors that must be considered include the breast cancer subtype, the extent of disease at the time of diagnosis, the mother’s general health, the proposed treatment plan, the health of the fetus and the gestational age at diagnosis [7].

Compared with breast cancer in nonpregnant patients, breast cancer in pregnant women is often diagnosed at a more advanced stage and is more likely to present with a large tumor size, high nuclear grade, lymph node involvement, lymphovascular invasion and hormone receptor–negative status [8]. The treatment goals for pregnant patients are the same as those for nonpregnant patients: to achieve local disease control and prevent systemic recurrences [9]. Pregnancy itself does not appear to be associated with worse breast cancer outcomes [10,11]. With appropriate local and systemic therapy, women treated with chemotherapy during pregnancy have clinical outcomes that are no worse than those of nonpregnant patients matched for age and breast cancer stage [12].

Treatment recommendations for pregnant patients have been based upon case reports, retrospective data, case series and anecdotal evidence [13]. In selected cases, surgery may be the first therapy offered. Chemotherapy, given either neoadjuvantly or adjuvantly, is often required because most pregnant patients are young and present with biologically aggressive and/or large locally advanced tumors. When radiation therapy for breast cancer is needed, it is deferred until after childbirth to avoid radiation exposure to the fetus.

Information on the effects of antineoplastic drugs administered during pregnancy has been focused largely on fetal abnormalities (for example, spontaneous abortion, malformations, fetal intrauterine growth retardation, fetal death). The period of pregnancy during which the fetus is exposed to chemotherapy appears to be critical, as the highest risk for fetal malformation occurs during fetal organogenesis in the first trimester [14]. When chemotherapy is administered during the second and third trimesters, the reported fetal malformation rates range from 1.3% to 3.8%, which are similar to the rate reported for the general population [15-17]. Anthracycline-based chemotherapy can be administered safely during the second and third trimesters [18,19]. There are also data accumulating that support the use of taxanes during pregnancy [20,21]. However, trastuzumab and endocrine therapy are reserved until after childbirth because of the risks of fetal complications, even when given in the second and third trimesters [16].

Although increasing evidence showing the safety and efficacy of treatment with chemotherapy for breast cancer in the second and third trimesters has emerged, the concurrent diagnosis of breast cancer and pregnancy remains a clinical and ethical challenge for the oncologist, obstetrician, pediatrician and patient. Limited data are available regarding the long-term outcomes of children exposed to chemotherapy *in utero*, and there are currently no specific guidelines for monitoring such children. More data about the long-term effects of gestational chemotherapy exposure are needed to guide pediatricians and other health care providers as they continue to care for children with such exposure. At the University of Texas MD Anderson Cancer Center, women with breast cancer diagnosed during pregnancy have received the same systemic chemotherapy regimen—5-flurouracil, doxorubicin and cyclophosphamide (FAC)—in the second and third trimesters since 1992 [13]. In 2006, Hahn *et al*. reported on the initial follow-up of children exposed to these drugs *in utero* and found no significant short-term complications for the majority of those children [18]. The objective of this study was to provide an update 8 years after the initial report on the acute neonatal complications and expand on the postneonatal outcomes of this cohort of children exposed to chemotherapy *in utero*.

## Methods

Between 1992 and 2010, 81 patients who provided informed consent and met the eligibility criteria for a prospective, institutional review board (IRB)–approved trial at our institution were treated using a standardized chemotherapy regimen during the second and third trimesters of their pregnancy. The IRB at the University of Texas MD Anderson Cancer Center provided approval for this study. Patients signed an IRB-approved informed consent to be eligible for the study. No separate ethics committee approved this study outside of the institutional review board. All enrolled patients were treated in compliance with the Declaration of Helsinki. The patients received outpatient combination chemotherapy (FAC) with cyclophosphamide (500 mg/m^2^ intravenously on day 1), doxorubicin (50 mg/m^2^ by continuous infusion over 72 hours) and two bolus doses of 5-fluorouracil (500 mg/m^2^ intravenously on days 1 and 4) [18]. Each cycle was given every 21 to 28 days, and therapy lasted through gestational week 35. Other standard systemic therapies, such as trastuzumab and endocrine therapy with tamoxifen, were given after childbirth. Outcomes of the women and short-term outcomes of the children exposed *in utero* have been reported previously (*n* = 57) [13,18].

As part of their participation in the registry trial for the treatment of breast cancer during pregnancy, the participants also provided consent for obtaining delivery records and subsequent questionnaires that provided data for this analysis. Labor and delivery records were obtained, and relevant data were recorded and updated in a prospective manner. A health survey/questionnaire (Table 1) was sent to the parents or guardians of the children who were exposed to chemotherapy *in utero* to evaluate immediate and long-term outcomes. The survey was divided into two portions: neonatal outcomes and postneonatal outcomes. The survey questions covered issues related to delivery, puberty, reproductive capacity and overall health concerns. The survey was added to the study protocol in 2001, and the resultant data were collected from 2001 through 2005. The survey was sent out a second time in 2010 in an effort to capture as much data as possible. The data are included in this article. Descriptive statistics were used to report the results.

**Table 1:** **Health questionnaire**

**Questionnaire items**	
**Delivery and current health status**	
1	Delivery statistics: weight, weeks gestation, C-section or vaginal delivery
2	Did your child have any medical problems at the time of delivery such as having fever, needing oxygen, or being admitted to a special care unit, such as intensive care?
3	Did your child have any medical problems in the first month after birth after being discharged home after delivery?
4	Do you consider your child to be healthy?
5	Does your child have any of the following:
	Breathing problems such as asthma
	Heart problems such as a heart murmur
	Stomach or other gastrointestinal problems such as heartburn
	Bone/skeletal problems such as arthritis
	Urinary problems (kidney or bladder problems)
	Neurological problems (numbness, weakness)
	Ear/nose/throat problems including hearing
	Allergies/eczema
	Eye/visual problems
**Developmental/social history**	
6	If in school, has your child had any difficulties in school such as requiring smaller class size, extra tutoring or one-on-one teaching?
7	Has your child started to have any developmental changes related to puberty?
8	Has your child become pregnant or fathered a child?
9	Has your child tried to conceive, but had difficulty?
**Concerns about cancer and parenthood**	
This section detailed the number of previous pregnancies at time of diagnosis, wishes for future pregnancies, Reproductive Concerns Scale and questions related to how pervasive the thoughts of cancer are in day-to-day life.	

## Results

Of the 81 participants, 63 patients or their next of kin (78%) answered at least some portion of the survey regarding the child exposed to chemotherapy *in utero*. Of the surveys not accounted for, seven participants were lost to follow-up. The reasons for inadequate follow-up included death of study participant, a preference not to respond or the inability to identify next of kin. Eleven participants were enrolled in the study after the second round of surveys was mailed in 2010. These participants who did not respond to the surveys were included in the labor and delivery data, as these data were available.

Eighty-one children were born after exposure to varying numbers of anthracycline-based chemotherapies *in utero*. Among the cohort, there were a total of 328 cycles of FAC administered (mean = 4.2 cycles) (*N* = 81). Eighty-six percent had exposure to at least four cycles of anthracycline-based chemotherapy. Delivery data and neonatal complications are shown in Tables 2, 3 and 4, respectively. The mean gestational age at delivery was 37 weeks (range, 29 to 41 weeks). The mean birth weight overall was 2.9 kg (range, 1.4 to 3.9 kg). Of the patients with information available regarding delivery, 33% were delivered by cesarean section, while 67% underwent normal vaginal delivery.

**Table 2:** **Delivery outcomes for children exposed to chemotherapy** ***in utero*** **, overall**

**Delivery outcome**	**Data**
Mean gestational age at delivery, wk	37 (29 to 41)
Mean birth weight, kg	2.9 (1.3 to 3.9)
Type of delivery, *n* (%)	
Cesarean section	27 (33.3)
Vaginal	54 (66.7)
Chemotherapy exposure, *n* (%)	
>4 cycles of FAC^a^	70 (86.0)
≤3 cycles of FAC	5 (6.0)
Unknown number of cycles	6 (7.0)

^a^FAC, 5-Fluorouracil, doxorubicin, and cyclophosphamide.

**Table 3:** **Delivery outcomes for children exposed to chemotherapy** ***in utero*** **, preterm**

**Delivery outcome**	**Result,** ***n***
Preterm births (<37 wk)	28
Late preterm (32 to 36 wk)	27
Early preterm (<32 wk)	1
Cesarean section delivery	11
Vaginal delivery	17

**Table 4:** **Neonatal complications of children exposed to chemotherapy** ***in utero***

**Complication**	***n*** **(%)**	**≥4 cycles of anthracycline-based** ***in utero*** **chemotherapy exposure,** ***n***	**<4 cycles of anthracycline-based** ***in utero*** **chemotherapy exposure,** ***n***	**Preterm,** ***n*** **(<37 wk)**	**Term,** ***n*** **(≥37 wk)**
Any neonatal complications at delivery (*N* = 63^a^)	21 (33)	13	8	14	7
Breathing difficulties	11 (17)	9	2	7	4
Subarachnoid hemorrhage	1 (2)	1	0	1	–
Jaundice	3 (5)	2	1	2	1
Low heart rate	2 (3)	1	1	1	1
Hypoglycemia	2 (3)	0	2	1	1
Abnormal temperature	2 (3)	2	0	1	1
NICU/hospitalizations (*N* = 63^a^)	9 (14)	5	4	4	5
Prolonged NICU stay^b^ (>1 mo)	2 (3)	0	2	2^c^	–
Temporary hospital or NICU stay (≤14 days)	7 (11)	5	2	2	5

^a^Information on complications was available for 63 births. ^b^The reason for prolonged neonatal intensive care unit (NICU) stay was prematurity. ^c^One child had a 2-month stay due to being born at 29 weeks. The other child was delivered at 35 weeks.

Twenty-eight children were delivered preterm, defined as prior to 37 weeks’ gestation. Of these, one child was delivered at 29 weeks, weighing 1.39 kg and requiring a prolonged neonatal intensive care unit stay lasting 2 months. Another child with exposure to one cycle of anthracycline-based chemotherapy *in utero* was born at 32 weeks with a weight of 3.29 kg and required supplemental oxygen at the time of delivery. The remaining 26 preterm births occurred during gestational weeks 33 to 36. Among the preterm births, the mean birth weight (2.8 kg) and the mean number of chemotherapy cycles exposed to *in utero* (four cycles) were similar to the those in the general cohort. The majority of the preterm births were spontaneous vaginal deliveries (*n* = 27; 63%), without complications. Eleven preterm births (40%) occurred by cesarean section, and two of the mothers required emergent procedures. One of the emergent procedures was associated with maternal heart problems and fetal distress, and the other was a breach delivery.

At delivery, 63 patients had data evaluable for neonatal complications. The following complications were noted: 17% (*n* = 11) required extra oxygen, 2% (*n* = 1) had subarachnoid hemorrhage, 3% (*n* = 2) had hypoglycemia and 5% (*n* = 3) had jaundice (Table 4). Of the 63 patients, 38 had been exposed to four or more cycles of chemotherapy *in utero*, and the majority (65%) did not have any neonatal complications documented. One child with six cycles of *in utero* chemotherapy exposure was born with poor tone, which resolved soon after birth (not shown in Table 4). Table 4 displays the neonatal complications documented according to the number of patients exposed to four or more cycles of *in utero* chemotherapy and according to whether the delivery was preterm or at term. Of the 21 patients with neonatal complications, 62% were exposed to at least four cycles of chemotherapy *in utero*. Seventeen percent of neonates had breathing difficulties at birth. Among the preterm neonates with respiratory difficulty, almost all the cases were due to premature lung development. These neonates required at most supplemental oxygen by nasal cannula. One preterm neonate did have documented respiratory distress syndrome. Of the term neonates, one had just nasal flaring and one had transient tachypnea requiring oxygen for 24 hours. Another term neonate had nuchal cord and required cardio-pulmonary resuscitation because of this and one other had labored breathing with supplemental oxygen and therefore was intubated but subsequently weaned off within 8 hours. Eighty-two percent of neonates with breathing difficulties and fifty-six percent requiring temporary or prolonged hospital stays had been exposed to four or more cycles of chemotherapy *in utero*. Of the entire cohort (*N* = 81), congenital abnormalities remained the same as reported in 2006 [18]: one child was born with Down syndrome, one with clubfoot and one with ureteral reflux (hydronephrosis).

Postneonatal outcomes are given in Table 5, which shows health conditions reported in the survey and the prevalence of these conditions in the general population. The median age of the children at the time of survey completion was 7 years (range, <1 to 21 years). Of the 50 participants who responded to the portion of the survey evaluating postneonatal outcomes, 13 (26%) were between 13 and 22 years old. The remainder had children who were school-aged (ages 3 to 12 years; *n* = 19) or infants (0 2 years of age; *n* = 17). The majority of survey responders considered their children to be healthy overall, and only minor health issues were reported, as noted in Table 5. Of note, 31 patients reported a health issue, and 61% of these 31 patients had extensive *in utero* chemotherapy exposure (four or more cycles). For each data point of the health issues reported, the prevalence in the general population has been noted in Table 5 as a reference for comparison as well as the number of those with each health concern who have been exposed to four or more cycles of anthracycline-based chemotherapy *in utero*. Nineteen children were described as being healthy, and 47% of these had *in utero* exposure to four or more cycles of anthracycline-based chemotherapy. Twelve percent of the survey responders indicated that their children had developmental milestone delays. Three of these were childhood language delays. There were no significant cognitive abnormalities reported in the children. Of the 37 children who were reported to be enrolled in preschool through college, four reported having any difficulties in school. Three children, one of whom had recently graduated from college, were reported to have reading delays, and one child in seventh grade was reported to have difficulty with attention span. Eleven children had advanced past the seventh grade, and the majority (90%; *n* = 10) reported no difficulties. There was one child enrolled at the time in a college bachelor’s degree program. Other health problems reported by survey responders included 18 children with allergies and/or eczema (36%), 5 with asthma (10%), 1 with migraine headaches and 1 with absence seizures. Only allergies and/or eczema occurred at a prevalence higher than the general population reference rate. Sixty-seven percent of those with allergies and/or eczema had been exposed to more than four cycles of anthracycline-based chemotherapy *in utero*, and four of the five children who developed asthma also had more extensive chemotherapy exposure *in utero*. Furthermore, of the five children diagnosed with asthma, three had been born preterm and had breathing difficulties secondary to prematurity at birth. Ear, nose, and throat (ENT) issues were reported in 8% of children. One child was reported to have a hearing deficit and had exposure to six cycles of chemotherapy *in utero*. The other ENT concerns included sinus problems and recurrent otitis media, with one of these individuals requiring a myringotomy. The majority of survey respondents informed us that their children had not experienced changes related to puberty; only 24% (*n* = 12) of children had encountered pubertal changes. It is important to note, however, that only 13 children were of pubertal age (age 13 years and older). The mean age of puberty onset was 11 years (range, 10 to 13 years), and only one child was noted to have precocious hair growth at age 4 years. There are not yet any offspring of the children.

**Table 5:** **Postneonatal outcomes of children exposed to chemotherapy** ***in utero***

**Reported outcome**	***n*** **/** ***N*** **(%)**	**≥4 cycles of anthracycline-based** ***in utero*** **chemotherapy exposure,** ***n*** **/** ***N*** **(%)**	**Population prevalence, % [reference]**
Child considered healthy	49/50 (98)	–	–
Developmental milestone delays	6/50 (12)	2/6 (33)	10 [22]
Difficulties in school^a^	4/37 (11)	3/4 (75)	6-11 [22]
Reported health concerns	31/50 (62)	19/31 (61)	–
Allergies/eczema	18/50 (36)	12/18 (67)	11 to 25 [22]
Asthma/breathing	5/50 (10)	4/5 (80)	13 [22]
Vision	8/50 (16)	5/8 (63)	14 [22]
Heart murmur^b^	1/50 (2)	–	1.3 [23]
“Lazy eye”	2/50 (4)	1/2 (50)	1.6 to 3.6 [24]
Absence seizures^c^	1/50 (2)	1/1 (100)	<1 [25]
ENT^d^	4/50 (8)	3/4 (75)	HL, <1; ROM, 18 to 26^d^ [26]
GERD^e^	2/50 (6)	2/2 (100)	5 to 67^f^ [27]

^a^Reading delay (*n* = 3) and difficulty with attention span (*n* = 1); data are for 37 school-aged children. ^b^Resolved by age 1 year. ^c^Possibly hereditary. ^d^Ear, nose, and throat (ENT) includes recurrent otitis media (ROM; *n* = 2), sinus problems (*n* = 1) and hearing deficit (HL; *n* = 1). ^e^Gastroesophageal reflux disease. ^f^Varies according to age.

## Discussion

Our data indicate that treating women with anthracycline-based systemic chemotherapy for breast cancer during the second and third trimesters of pregnancy can be administered without significant impairment of the health of their offspring at delivery or into childhood compared with children in the general population. To date, limited data exist regarding potential health concerns for children exposed to chemotherapy *in utero*. Hahn *et al*. reported in 2006 on the presentation, treatment and outcomes of the first 57 pregnant women treated according to study protocol. The authors reported the outcomes of children who were exposed to chemotherapy *in utero*; and they found the majority of the children to be healthy and without significant developmental problems [18]. The German Breast Group enrolled 413 pregnant women with early-stage breast cancer in a prospective registry study and found that the statistically insignificant increased incidence of low birth weights and obstetrical complications observed was linked to premature delivery rather than to *in utero* chemotherapy exposure during the second and third trimesters [19]. Furthermore, Amant *et al*. investigated children who were prenatally exposed to any maternal cancer staging and treatment, including chemotherapy, and found no impairment in the general health or growth of these children. Specifically, there were no associations with increased central nervous system, cardiac or auditory toxicity [28].

In the present study, we found that, at a median age of 7 years, children who were exposed to anthracycline-based chemotherapy *in utero* were growing well, with no significant toxic effects observed related to chemotherapy exposure. There were three children born with congenital abnormalities, which were Down syndrome, ureteral reflux and clubfoot. The child with Down syndrome was born to a 32-year-old woman. The rate of congenital abnormalities in our population is similar to the US average rate of approximately 3% [15]. The chief long-term health concerns were allergies and/or eczema, which occurred more commonly in the children in our study than currently reported in the general population. The majority of patients reporting allergies and/or eczema were exposed to four or more cycles of anthracycline-based chemotherapy *in utero*; however, this finding may be the result of generalized overreporting. There have been no significant delays reported in puberty. To determine whether more extensive *in utero* chemotherapy exposure translates to worse health outcomes overall will require more prolonged follow-up and a prospective study with more objective data. It is reassuring that in the current era, most women are likely to receive four cycles of FAC in total, with perhaps less being given during the *in utero* period, depending on the timing of the diagnosis of breast cancer during the pregnancy and the timing of the surgical intervention. Continuing to follow cohorts of children such as these, along with multi-institutional and multinational collaborations, are needed to further address these concerns for the children, especially as they mature into adults.

Our results support previous efforts to evaluate whether children of cancer patients encounter any late adverse health effects due to *in utero* chemotherapy exposure. In the 457 studies reviewed in the National Toxicology Program monograph (2013) [29], 60% of the children exposed to gestational chemotherapy had follow-up data available. Normal growth and development were reported for all; however, most of the children were not evaluated beyond year 2 of life. Other investigators have performed longer-term evaluations of children exposed to chemotherapy *in utero*. The largest dataset to date consists of 84 pregnant women who were treated for hematologic malignancies and whose children and grandchildren (*n* = 12) participated in a study for follow-up of the long-term effects of the treatment. At a median follow-up of 18.7 years, there were no cases of leukemia and no long-term ill effects related to physical health, growth or development among the children exposed to chemotherapy *in utero* or among their offspring [30].

The main limitations of the present study include its small sample size, subjective nature (via questionnaire) and short follow-up period. The numbers of older and postpubertal children are small. The outcomes of the exposed children were assessed through a survey distributed to the children’s parents or guardians. Although the response rate for this study was satisfactory, we could not assess all eligible children. Behavioral and emotional outcomes of the children might be better addressed by a survey directed to the children themselves. Although the survey form was written at a low literacy level, the educational backgrounds of the respondents may have varied and thus may have contributed to the number of incomplete surveys. Additionally, it might be effective to minimize free text and create a standardized case report form [19] for data collection, which could be expanded to capture data from other cohorts in centers across the United Stataes. Furthermore, the results may have been more reliably gained by using an objective format such as pediatricians’ records or chart reviews as compared to questionnaires. It will be important to continue to follow these patients in the future and obtain more objective data for them, such as records from pediatric visits and hospitalizations. Additionally, the follow-up period was short, as the median age of the children was 7 years. Continued follow-up of this growing cohort is essential. It will be valuable to note the incidence of chronic health conditions, cancer and fertility issues as the children enter puberty and adulthood and of labor and/or delivery complications as the children in the study have children of their own.

## Conclusions

This cohort of children exposed to chemotherapy *in utero* appeared to be doing well, with no trend to indicate a higher rate of serious medical problems than that seen in the general population. This is an important prospective single-institution study of outcomes of children exposed *in utero* to anthracycline-based chemotherapy for breast cancer. Pregnant women and physicians can be reassured that treating breast cancer during the second and third trimesters with anthracycline-based chemotherapy does not jeopardize the health outcomes of the developing fetus. No specific recommendations regarding health monitoring for these children outside of the recommendations for the general population are supported by these data. Potential concerns may arise and will need to be followed in this cohort as well as others. These concerns include future childbirth and fertility issues as more offspring reach puberty, as well as the cancer incidence in the population exposed to chemotherapy *in utero*. Future exploration of cognitive impairment, high school graduation rates and college attendance rates, as well as continued long-term follow-up of this and other cohorts, is needed to provide more information to parents and pediatricians.

## Abbreviations

ENT: Ear, nose and throat
FAC: 5-Fluorouracil, doxorubicin, and cyclophosphamide
GERD: Gastroesophageal reflux disease
IRB: Institutional review board
NICU: Neonatal intensive care unit
